# Urban–Rural Disparities in Food Insecurity and Weight Status among Children in the United States

**DOI:** 10.3390/nu16132132

**Published:** 2024-07-04

**Authors:** Jayna M. Dave, Tzuan A. Chen, Alexandra N. Castro, Mamie A. White, Elizabeth A. Onugha, Sloane Zimmerman, Debbe Thompson

**Affiliations:** 1 USDA/ARS Children’s Nutrition Research Center, Department of Pediatrics, Baylor College of Medicine, 1100 Bates Avenue, Houston, TX 77030, USA; alexandra.castro@bcm.edu (A.N.C.); mawhite@bcm.edu (M.A.W.); dit@bcm.edu (D.T.); 2 Department of Psychological, Health, and Learning Sciences, University of Houston, 3657 Cullen Boulevard, Houston, TX 77204, USA; tchen3@central.uh.edu; 3 HEALTH Research Institute, University of Houston, 4349 Martin Luther King Boulevard, Houston, TX 77204, USA; 4 Renal Services, Texas Children’s Hospital, 1102 Bates Avenue, Houston, TX 77030, USA; eaonugha@texaschildrens.org; 5 Department of Pediatrics—Nephrology, Baylor College of Medicine, 1102 Bates Avenue, Houston, TX 77030, USA; 6 Department of Pediatrics—Gastroenterology, Baylor College of Medicine, One Baylor Plaza, Houston, TX 77030, USA; sloane.zimmerman@bcm.edu

**Keywords:** food insecurity, weight status, urban–rural differences, children, NHANES

## Abstract

Place of residence (urban versus rural) is a contextual determinant of health that has received less attention in the food insecurity literature. The purpose of this study was to assess the urban–rural disparity in the prevalence of food insecurity and weight status among US children. Using data from the National Health and Nutrition Examination Survey (NHANES) 2013–2016 with three age groups of children (2–5, 6–11, and 12–17 years old), the associations of weight status and child and household food security status by urban–rural residence were examined using Rao–Scott Chi-square tests. Statistical significance was set at *p* < 0.05. Children living in urban areas were significantly more likely to experience household food insecurity (29.15%) compared to their rural counterparts (19.10%), among those aged 6–11 years. The associations between children’s weight status and child and household food security status were significant for children living in urban areas overall and different age groups but not for children living in rural areas. These trends were more pronounced in older age groups. Given the link between food insecurity and higher obesity rates, particularly among urban children, this study highlights the importance of incorporating food security interventions into future obesity prevention programs.

## 1. Introduction

Childhood obesity is a complex issue with far-reaching consequences. Identifying the behavioral, social, and environmental factors that contribute to it, such as access to healthy foods and the influence of marketing, is essential for designing effective prevention and treatment strategies [1,2]. Living in rural areas may be a risk factor associated with childhood obesity. A meta-analysis of 10 studies found that children in rural areas had a 26% greater risk of obesity than urban children [3]. Similar findings were observed in children and adolescents, with one study reporting greater odds of obesity in rural/nonmetropolitan areas compared to urban/metropolitan areas [4,5,6].

Prior research consistently shows a high prevalence of overweight and obesity among children living in rural communities in the US. A study in Mississippi found that 54% of middle school children exceed healthy weight ranges [7]. Similarly, a study in Appalachian Kentucky reported that 33% of elementary school children had a BMI over the 85th percentile [8], indicating a risk of obesity. These findings align with studies on African American children from rural South Carolina [9] and fourth-graders in rural Iowa [10]. While these studies show a consistent pattern, they often focus on specific regions or populations. To gain a broader understanding, data from nationally representative samples of both urban and rural children obtained simultaneously are crucial. Data from the National Survey of Children’s Health support the observed pattern and reveal a higher prevalence of overweight and obesity among children living in rural areas than their urban counterparts (35% vs. 30%) [11]. Additionally, two studies utilizing nationally representative samples [6,12] found significantly higher obesity rates among rural children compared to urban children. These findings strengthen the association between rural residency and childhood obesity.

While the link between rural residency and childhood obesity appears clear, the picture becomes more complex when considering food insecurity, which is the limited or uncertain availability of nutritionally adequate and safe foods and is strongly related to poverty [13]. The overall rates of food insecurity in the United States (US) in 2022 were significantly higher than the national average (12.8%) for households with children (17.3%) and households in principal cities (urban—15.3%) and rural areas (14.7%) [13]. Among the 17.3% of the food-insecure households with children, 8.6% of adults experienced food insecurity, while the children themselves were food-secure [13]. In a concerning 8.8% of the households, both children and adults suffered food insecurity at some point during the year [13]. Very low food security, the most severe level, affected both children and adults in approximately 1% (381,000 households) of US households with children [13]. Moreover, rates of food insecurity are highest in urban cities (15.3%), followed by rural areas (14.7%), and are lowest in suburban areas (10.5%) [13].

Research on the association between food insecurity and obesity in low-income children has yielded mixed results. Some studies, including one with a national sample of low-income children 8–17 years of age and another with young preschoolers (predominantly Hispanic) receiving Woman, Infants, and Children (WIC) services, found no association between overweight/obesity and household food insecurity [14,15]. However, other studies, such as one with a national sample of children 12–18 years of age and a longitudinal study of low-income preschool children in the Massachusetts WIC program, reported that children from food-insecure households were significantly more likely to be overweight than their counterparts from food-secure households [16]. These contrasting findings highlight the complexity of the relationship between food insecurity and obesity, particularly among low-income children.

With the increasing rates of both obesity and food insecurity in the US [13,17,18], examining the association between these issues among children is necessary. Thus, the purpose of this study was to investigate how food insecurity and weight status vary between urban and rural areas among US children, utilizing a nationally representative sample. This ongoing research will be crucial for developing effective interventions that address the diverse challenges faced by children across different geographic locations.

## 2. Materials and Methods

### 2.1. Participants

This study analyzed data from the National Health and Nutrition Examination Survey (NHANES) conducted between 2013 and 2016. Because the data were publicly available, they were determined to be exempt from review by the Baylor College of Medicine’s Institutional Review Board. NHANES, a cross-sectional survey representing the civilian non-institutionalized US population, is conducted by the National Center for Health Statistics (NCHS) of the Centers for Disease Control and Prevention (CDC). Information about the survey methodology and sampling design is available from other sources [19]. We used data from two survey cycles (2013–2014 and 2015–2016) that included information on food security and weight status to increase the reliability and stability of estimates across subgroups [19]. The study focused on children aged 2 to 17 with reported food security status. Children with missing data on key variables were excluded from the analysis. A total of 6403 children were included, with children categorized into three age groups: 2–5 years old; 6–11 years old, and 12–17 years old. Following approval by the US CDC’s National Center for Health Statistics (NCHS) Ethics Review Board and after obtaining written informed consent from participants, the NHANES protocols ensured ethical data collection.

### 2.2. Measures

#### 2.2.1. Sociodemographic Variables

The NHANES gathered information on participants’ backgrounds through in-home interviews using a computerized system. Individuals 16 years old or older and those considered legally independent (emancipated minors) were interviewed directly. For children under 16, a designated person (proxy) familiar with their situation provided the information. The interviews captured details on age, gender, race/ethnicity, parent’s marital and education status, and household income. Data on participation in federal nutrition assistance programs such as the Supplemental Nutrition Assistance Program (SNAP), WIC, School Breakfast Program (SBP), National School Lunch Program (NSLP), and Summer Food Service Program (SFSP) were also collected.

To understand participants’ living environments, NHANES assigned a rural–urban status based on their county of residence. This classification relied on the Urban Influence Code (UIC), established by the United States Department of Agriculture (USDA)’s Economic Research Service (ERS). The 2013 Area Resource File, a comprehensive database of all 2142 US counties, was used to select the county codes for this study. Counties with UICs of 1 or 2 were categorized as urban. The USDA ERS designates all other codes (3–12) as nonmetropolitan codes, which were classified as rural in this study [20,21]. It is important to note that county-level urban–rural status is considered restricted data in NHANES. Access to these data was granted by the NCHS Research Data Center (RDC).

#### 2.2.2. Food Security Status

The standardized 18-item US Household Food Security Survey Module was used during the NHANES in-home interview to assess food security status [22]. An adult in the household answered 10 general questions about the entire household. If children under 18 were present, the adult also answered an additional 8 questions specifically about the children’s experiences. Focusing on the past 12-month period, the survey included questions on food security status (household and child level), with responses coded using the USDA’s coding guide. A score of 0–18 was calculated by adding the affirmative responses. Higher scores indicate greater food insecurity. Following established guidelines [23], children in the household were classified as food-insecure if the adult answered with two or more affirmative responses to the child-specific questions. NHANES categorizes food security into four levels: high, marginal, low, and very low [24]. For this study, we combined “high” and “marginal” into one category of food security; similarly, “low” and “very low” were combined into one category of food insecurity.

#### 2.2.3. Weight Status

As part of NHANES, height and weight data for the participating children were also collected using standardized procedures [25]. We used body mass index (BMI) to assess weight status in the children included in this study. BMI is a common method that calculates weight in kilograms divided by height in meters squared (kg/m^2^). Following the CDC’s 2000 sex-specific BMI-for-age growth charts [26], weight status was categorized as underweight (below 5th percentile), normal weight (5th to <85th percentile), overweight (85th to 95th percentile), and obese (≥95th percentile). It is important to note that due to the small sample size for underweight children, this study combined their data with those of the normal-weight group for analysis.

### 2.3. Data Analysis

The descriptive statistics of the participants’ characteristics were calculated for three age groups (2–5, 6–11, and 12–17 years old) representing early childhood, childhood, and adolescence. We examined the associations between participant characteristics and age groups. For continuous variables, we employed ANOVA, while Rao–Scott Chi-square tests were used for categorical variables. To account for the NHANES complex survey design, including non-response, and stratification adjustments, sample weights were incorporated into the analyses.

To ensure that national representative estimates will be generated, the SAS SURVEYFREQ procedure, which accounts for the complex, stratified, multistage probability cluster sampling design, was used, as specified in the instructions for using NHANES data [27]. The associations of weight status and child/household food security status by different urban–rural residence and age groups were examined using Rao–Scott Chi-square tests. All analyses were performed using SAS 9.4. Statistical significance was set at *p* < 0.05.

## 3. Results

The overall sample of 6403 children included a balanced distribution of age groups—27.33% were 2–5 years old, 40.43% were 6–11 years old, and 32.23% were 12–17 years old (Table 1). Just over half (51%) of the children were male, and the remaining 49% were females, with a slight majority of Hispanic children (30.97–33.80%). The majority of children were classified as normal weight (59.03–71.95%) across each of the age groups. High participation rates in federal nutrition assistance programs were observed (~89% in SNAP and ~90% in school meals programs including SBP and NSLP).

Children aged 2–5 years old experienced the lowest rates of food insecurity (9.66%) compared to older age groups, which rose to 14.77% among 12–17-year-olds; household food insecurity ranged from 26.19% among 2–5-year-olds to 29.42% in 12–17-year-olds. These can be categorized as moderate rates of food insecurity.

The associations between children’s weight status and child food security status were significant for children living in urban areas overall and for different age groups (*p* < 0.05) (Table 2). However, the associations were not significant for children living in rural areas. Underweight/normal-weight children from urban areas were more likely to report experiencing food security; however, overweight or obese children from urban areas were more likely to be in households with food insecurity. These trends were more pronounced in older age groups of children. Similar significant trends were also found for household food security among children in urban areas (Table 3). Children living in rural areas also showed similar non-significant trends in child and household food insecurity by age group.

## 4. Discussion

This study addresses a critical gap by examining the complex interplay between children’s weight status, food security, and their residential environment (urban vs. rural) across different age groups. It delves deeper into exploring these associations across different age groups, a unique contribution compared to previous research.

For the overall sample, the child and household food insecurity rates were categorized as moderate. While these rates may seem statistically moderate, it is crucial to remember that food insecurity exists on a spectrum. Even moderate levels can negatively impact children’s health and well-being through nutrient deficiencies, impaired cognitive development, and an increased risk of chronic diseases like obesity and diabetes [28,29]. By acknowledging the multifaceted consequences of even moderate food insecurity, we can emphasize the importance of addressing this issue and ensuring all children have access to a safe and reliable food supply.

This study identified a noteworthy urban paradox. Children 6–11 years old reported higher household food insecurity compared to their rural counterparts. Moreover, in urban areas, significant associations emerged between weight status and both child and household food security for all age groups of children. Underweight and normal-weight children were more likely to be food-secure, while their overweight or obese counterparts faced a higher likelihood of food insecurity. This aligns with some studies suggesting limited access to healthy and affordable food options in urban areas, particularly low-income neighborhoods [30,31,32]. Children in these situations might experience food insecurity while simultaneously having easier access to calorie-dense, less nutritious options, contributing to obesity.

The observation that these trends strengthened with increasing age suggests a cumulative effect of unhealthy food environments. As children in urban areas age, the challenges associated with accessing healthy food and navigating unhealthy food marketing may become more pronounced, potentially leading to a stronger association between weight status and food insecurity. These results highlight the unique challenges faced by urban children in achieving optimal nutrition. Similar observations on the cumulative effect of unhealthy food marketing exposure in urban environments have been documented in other studies [33,34,35,36,37]. Conversely, no significant associations were observed in rural settings.

There are several explanations for these findings. Urban environments, particularly low-income areas with limited access to healthy foods, often grapple with higher poverty rates [32,38,39,40,41]. This translates to increased food insecurity and childhood obesity rates in urban areas [42]. Additionally, exposure to unhealthy food marketing in urban environments may contribute to unhealthy consumption patterns, ultimately leading to weight gain and obesity [35,36]. Moreover, there are social and cultural factors that contribute to the association between food insecurity and childhood obesity in urban areas [43,44,45]. For example, children living in neighborhoods with a high prevalence of obesity may be more susceptible to social norms that encourage unhealthy eating habits.

The associations observed in urban areas were not significant in rural settings. This disparity suggests that factors influencing children’s weight status and food security may differ substantially between rural and urban environments [46]. While urban areas may experience disparities related to socioeconomic status, access to food resources, and dietary behaviors, these disparities may manifest differently or to a lesser extent in rural areas. Understanding these differences is crucial for developing effective and targeted strategies to promote child nutrition and food security across diverse geographic locations.

These findings have important implications for public health and policy efforts. Tailored interventions are required to address the specific challenges associated with childhood obesity and food insecurity in urban areas. These initiatives should consider age-specific dynamics, as the age-food security link strengthens with age. Potential interventions could include educational programs on nutrition, cooking classes, and increased opportunities for physical activity in schools and communities. In contrast, rural areas may require a different approach, recognizing the unique factors that influence child weight status and food security in those settings. The findings also suggest that there is a need to focus on improving access to healthy and affordable foods in urban areas, potentially through initiatives like farmers’ markets and community gardens. Policy efforts in urban environments could also focus on regulating unhealthy food marketing and advertising directed toward children.

Further research is needed to gain a deeper understanding of the complex relationship between food security and weight status in children, particularly within urban settings. Future research should (i) focus on identifying the specific factors driving the association between food insecurity and childhood obesity in urban areas, which will be essential for informing the development of targeted interventions; (ii) investigate the long-term health and well-being impacts of food insecurity and childhood obesity; and (iii) further explore the social and cultural factors influencing the weight–food security link in urban environments. This would help to raise awareness of the importance of addressing these issues for a child’s overall health and development. This awareness can lead to stronger advocacy efforts and the allocation of resources for programs that address these critical issues for children’s nutritional well-being in diverse communities.

This study benefits from several strengths. First, it utilizes a large, nationally representative sample from the US population. This robust sample size allows for reliable conclusions to be drawn about the associations between child weight status, food security (both child and household), and residential environment (urban vs. rural). Second, this study leverages the strengths of NHANES, which employs well-established and reliable measures for anthropometric data (weight and height) through standardized protocols. One limitation of this study is its cross-sectional design. While it can identify associations between variables, it cannot establish cause-and-effect relationships. Other limitations are that this study did not explore potential sex or race differences in the associations of interest and did not control for participants’ characteristics such as gender, race/ethnicity, parent’s marital status and education status, household income, etc. Also, levels of physical activity were not investigated. This study categorized locations as urban or rural, but future research could benefit from further stratification within urban areas, such as distinguishing between central cities and suburbs, to capture potentially finer-grained variations in food insecurity and weight status. These are important factors that can influence both food security and weight status, and future research should explore how these demographics may influence the observed relationships. Lastly, the use of NHANES 2013–2016 data, while appropriate for investigating the core relationships of interest, represents a timeframe nearing ten years. Future research utilizing more recent NHANES cycles could provide valuable insights into potential changes in prevalence and how these associations may evolve over time.

## 5. Conclusions

This study reveals a critical urban–rural disparity in how children’s weight status relates to their experience with food insecurity. We observed a concerning trend in urban areas, where children experiencing food insecurity were more likely to be overweight or obese across all age groups. Notably, the strength of the association and how it varies by age were unique to urban environments. These findings highlight the need for tailored interventions and policies that consider both geographic location and age to effectively address children’s nutritional well-being and food security. In contrast, rural settings displayed no significant associations. This disparity highlights the importance of geographically specific strategies to address the distinct challenges faced by children in different environments. Further research is crucial to identify the underlying factors contributing to these disparities in urban areas. By understanding these factors, we can develop targeted interventions to mitigate the negative effects of food insecurity on children’s weight status. Ultimately, such research can inform the creation of programs and policies that promote healthy eating habits, improve access to nutritious food, and ensure food security for all children, regardless of their location or age.

## Figures and Tables

**Table 1:** Descriptive statistics of participant characteristics by age groups.

	All *n* = 6403	2–5 Years Old *n* = 1750	6–11 Years Old *n* = 2589	12–17 Years Old *n* = 2064
	Mean ± SD			
Age	7.51 ± 5.25	3.38 ± 1.14	8.44 ± 1.72	14.44 ± 1.68
BMI Percentile	19.81 ± 5.44	16.49 ± 1.85	18.79 ± 4.31	23.84 ± 6.19
	*n* (%)			
Gender				
Male	3266 (51.01)	903 (51.60)	1311 (50.64)	1052 (50.97)
Female	3137 (48.99)	847 (48.40)	1278 (49.36)	1012 (49.03)
Weight Status				
Normal weight	3893 (63.70)	1172 (71.95)	1547 (62.05)	1174 (59.02)
Overweight	1048 (17.15)	250 (15.35)	431 (17.29)	367 (18.45)
Obese	1170 (19.15)	207 (12.71)	515 (20.66)	448 (22.52)
Ethnicity				
Non-Hispanic White	1730 (27.02)	486 (27.77)	698 (26.96)	546 (26.45)
Non-Hispanic Black	1556 (24.30)	432 (24.69)	632 (24.41)	492 (23.84)
Hispanic	2111 (32.97)	542 (30.97)	875 (33.80)	694 (33.62)
Others	1006 (15.71)	290 (16.57)	384 (14.83)	332 (16.08)
Child Food Security Status				
Child food security	5480 (87.08)	1552 (90.34)	2202 (86.35)	1726 (85.23)
Child food insecurity	813 (12.92)	166 (9.66)	348 (13.65)	299 (14.76)
Household Food Security Status				
Household food security	4535 (72.04)	1268 (73.81)	1837 (72.01)	1430 (70.58)
Household food insecurity	1760 (27.96)	450 (26.19)	714 (27.99)	596 (29.42)
Participation in Federal Nutrition Assistance Programs				
SNAP	2129 (89.68)	673 (90.21)	875 (89.74)	581 (88.97)
WIC	479 (49.33)	479 (49.33)	NA	NA
SBP	2143 (92.57)	187 (94.92)	1253 (92.95)	703 (91.30)
NSLP	2606 (87.66)	218 (92.77)	1407 (88.60)	981 (85.30)
SFSP	640 (34.32)	49 (35.51)	354 (34.40)	237 (33.95)
Any of the FNAP	3722 (72.60)	938 (77.78)	1619 (71.83)	1165 (69.89)

SD: standard deviation; BMI: body mass index; SNAP: Supplemental Nutrition Assistance Program; WIC: Special Supplemental Nutrition Program for Women, Infants, and Children; SBP: School Breakfast Program; NSLP: National School Lunch Program; SFSP: Summer Food Service Program; FNAP: Federal Nutrition Assistance Program.

**Table 2:** Urban–rural differences in child food security status by weight status of children in different age groups.

	Urban			Rural		
Age Group	Food-Secure	Food-Insecure		Food-Secure	Food-Insecure	
	Weighted %		*p*-Value	Weighted %		*p*-Value
All			<0.0001			0.5475
Underweight/Normal weight	59.91	6.05		55.51	6.78	
Overweight	14.23	1.88		17.12	1.52	
Obese	15.08	2.83		16.54	2.53	
2–5 years old			0.0005			0.401
Underweight/Normal weight	68.44	5.36		69.13	5.82	
Overweight	12.82	1.49		13.62	0.51	
Obese	10.09	1.79		10.57	0.35	
6–11 years old			0.0478			0.5357
Underweight/Normal weight	58.21	6.94		57.45	6.72	
Overweight	14.11	2.22		15.58	0.83	
Obese	15.81	2.69		17.41	1.99	
12–17 years old			0.0001			0.7415
Underweight/Normal weight	56.21	5.62		47.76	7.25	
Overweight	15.24	1.80		19.94	2.52	
Obese	17.51	3.63		18.57	3.95	

**Table 3:** Urban–rural differences in household food security status by weight status of children in different age groups.

	Urban			Rural		
Age Group	Food-Secure	Food-Insecure		Food-Secure	Food-Insecure	
	Weighted %		*p*-Value	Weighted %		*p*-Value
All			<0.0001			0.3003
Underweight/Normal weight	52.23	13.74		50.82	11.47	
Overweight	11.89	4.22		15.77	2.87	
Obese	12.32	5.60		14.42	4.65	
2–5 years old			0.0059			0.8454
Underweight/Normal weight	58.44	15.37		62.12	12.83	
Overweight	10.34	3.97		12.15	1.98	
Obese	8.40	3.48		9.53	1.38	
6–11 years old			<0.0001			0.5152
Underweight/Normal weight	51.192	13.96		54.19	9.98	
Overweight	12.377	3.96		14.20	2.22	
Obese	12.697	5.81		14.85	4.55	
12–17 years old			<0.0001			0.4422
Underweight/Normal weight	49.346	12.50		42.98	12.03	
Overweight	12.384	4.64		18.67	3.79	
Obese	14.414	6.72		16.30	6.21	

## Data Availability

For data analysis, we used publicly available data, which can be accessed at https://wwwn.cdc.gov/nchs/nhanes/continuousnhanes/default.aspx?BeginYear=2013 (accessed on 31 January 2019).
